# International collaborative experiences in managing pyoderma gangrenosum in patients with inflammatory bowel disease

**DOI:** 10.1177/17562848261461012

**Published:** 2026-06-29

**Authors:** Jan Guse, Andreas Blesl, Philip Esters, Katja Matthes, Renate Schmelz, Carsten Schmidt, Julia Wanzl, Elisabeth Schnoy, Niels Teich, Viktoria Hentschel, Andreas Stallmach, Jessica Rueddel, Kathleen Lange

**Affiliations:** Department of Internal Medicine IV (Gastroenterology, Hepatology and Infectious Diseases), Jena University Hospital, Jena, Germany; Division of Gastroenterology and Hepatology, Department of Internal Medicine, Medical University of Graz, Graz, Austria; Department of Medicine I, Agaplesion Markus Hospital, Frankfurt, Germany; Medical Clinic and Polyclinic I, Carl Gustav Carus University Hospital, Technical University Dresden, Dresden, Germany; Medical Clinic and Polyclinic I, Carl Gustav Carus University Hospital, Technical University Dresden, Dresden, Germany; Medical Clinic II, Hospital Fulda, Fulda, Germany; Faculty of Medicine, Friedrich Schiller University Jena, Jena, Germany; 3rd Medical Clinic, University Hospital Augsburg, Augsburg, Germany; 3rd Medical Clinic, University Hospital Augsburg, Augsburg, Germany; Faculty of Medicine, Friedrich Schiller University Jena, Jena, Germany; Group Practice for Digestive and Metabolic Diseases Leipzig, Leipzig, Germany; Clinic of Internal Medicine I, University Hospital Ulm, Ulm, Germany; Department of Internal Medicine IV (Gastroenterology, Hepatology and Infectious Diseases), Jena University Hospital, Jena, Germany; Department of Internal Medicine IV (Gastroenterology, Hepatology and Infectious Diseases), Jena University Hospital, Jena, Germany; Department of Internal Medicine IV (Gastroenterology, Hepatology and Infectious Diseases), Jena University Hospital, Am Klinikum 1, Jena D-07747, Germany

**Keywords:** EIM, IBD, pyoderma gangrenosum

## Abstract

Pyoderma gangrenosum (PG) is a rare but challenging extraintestinal manifestation (EIM) of inflammatory bowel diseases (IBD), affecting 6%–48% of IBD patients. This retrospective study analyzed affected patients and evaluated therapeutic strategies for both IBD remission and PG resolution. A multicenter retrospective analysis was conducted on patients with IBD and PG in eight tertiary IBD centers. Demographic data, prior therapies, surgeries, and treatment of PG were collected retrospectively, and treatment responses were assessed. The cohort included 50 patients (median age: 43 years; 68% female). Crohn’s disease was present in 58% and ulcerative colitis in 42%. Fifty percent of patients had prior surgery, 68% had an intestinal stoma in their medical history. 48% were experienced with biologic therapy, predominantly anti-tumor necrosis factor (TNF) therapy (83%). PG mainly affected the lower extremities (52%) and peristomal areas (24%). Systemic steroids were used in 52% (26/50) and led to PG resolution in only 11.5% (3/26). Anti-TNF therapy was the main approach, used in 68% (34/50) of patients, with resolution achieved in 80% (27/34). Calcineurin inhibitors were given to 26% (13/50) of patients and induced resolution in 38% (5/13). Three of six non-responders were successfully switched to infliximab. Overall, PG resolution was achieved in 80% (40/50), correlating with IBD remission in 78% (31/40) of these patients. The median time to PG resolution was 4 months. Anti-TNF therapy was an effective treatment for PG in IBD patients, even in those with prior nonresponse to calcineurin inhibitors. Systemic steroids showed low response rates. PG healing mostly aligned with IBD remission, underlining the need for tailored long-term therapy.

## Plain language summary

Working together across countries to treat pyoderma gangrenosum in people with inflammatory bowel disease

Pyoderma gangrenosum (PG) is a rare and painful skin disease that can develop in people with inflammatory bowel disease (IBD). It often leads to slow-healing wounds and can strongly affect quality of life. In our study of 50 patients treated at specialized centers, pyoderma gangrenosum healed in most patients, particularly when the underlying bowel disease was well controlled. Anti-inflammatory treatments, most commonly biologic therapies—predominantly TNF-α antagonists—and systemic steroids were used; however, systemic steroids were associated with low response rates. In most patients, improvement of the skin disease occurred at the same time as improvement of IBD. This highlights the importance of coordinated, long-term treatment strategies that address both the bowel disease and its skin complications.

## Introduction

Inflammatory bowel diseases (IBD), including Crohn’s disease (CD) and ulcerative colitis (UC), are immune-mediated disorders of the gastrointestinal tract. Their pathogenesis involves a disrupted intestinal barrier and altered microbiome in genetically predisposed individuals, triggered by environmental factors. Approximately 0.5% of individuals in Western countries are affected, with global prevalence steadily increasing. By 2030, up to 1% of the world population may be affected, resulting in reduced quality of life, substantial social burden, and significant healthcare costs.^1^

Extraintestinal manifestations (EIM) are inflammatory conditions occurring outside the gastrointestinal tract as a consequence of systemic inflammation in IBD. Up to 50% of patients develop at least one EIM during the disease course, and one-third experience more than one EIM.^2^ Data on EIM in IBD remain limited and highly heterogeneous. In 2017, a systematic review assessing the use of biologics for EIM treatment failed to perform a meta-analysis due to low study numbers, variable quality, and inconsistent EIM definitions and outcomes. The authors concluded that EIM treatment represents a significant unmet clinical need.^3^

The German S3/ECCO guideline reports an EIM incidence of 20%–40% in patients with CD, with prevalence increasing with disease duration.^4^ In CD, high disease activity, long disease duration, female sex, and older age appear to favor EIM occurrence. In UC, disease extension beyond the left-sided colon is associated with an increased EIM risk.^5^ In a Swiss cohort, active disease and a positive family history of IBD were linked to persistent EIM in CD patients.^6^

Pyoderma gangrenosum (PG) is a rare neutrophilic dermatosis characterized by painful, rapidly progressive ulcerations, often triggered by minor trauma (pathergy). More than 50% of PG patients have an associated systemic inflammatory disease, and PG is frequently linked to IBD. The estimated prevalence of PG in IBD ranges from 0.4% to 2.6%, and in approximately 15% of cases, PG precedes the IBD diagnosis.^7^ PG is often associated with other EIM, permanent stoma formation, and female sex.^8^

The pathogenesis of PG remains incompletely understood, although dysregulated immune responses and neutrophil dysfunction are considered central mechanisms.^9^ PG represents a reactive cutaneous EIM sharing pathophysiological pathways with IBD, in contrast to cutaneous IBD manifestations with identical histology (e.g., metastatic CD), IBD-associated skin diseases (psoriasis, hidradenitis suppurativa, atopic dermatitis), or treatment-induced skin lesions.^2,10^

Management of PG, particularly in patients with IBD, is challenging due to its unpredictable course and high recurrence rates.^11^ Therapeutic options include systemic corticosteroids, cyclosporine, and biologic agents such as tumor necrosis factor (TNF)-α inhibitors and interleukin (IL)-12/23 inhibitors. However, owing to the rarity of PG, evidence is limited to a single randomized controlled trial,^12^ with most recommendations based on case reports, small case series, and expert opinion.^13,14^ Consequently, treatment often requires multiple therapeutic attempts, imposing substantial physical, psychological, and economic burden. The absence of validated selection criteria for individualized therapy, together with the clinical heterogeneity of PG, hampers treatment standardization and underscores the need for real-world evidence.

This multicenter retrospective study analyzes treatment responses in patients with PG associated with IBD. By systematically evaluating therapeutic outcomes across multiple institutions, we aim to characterize the effectiveness of different treatment regimens and contribute evidence to support improved clinical decision-making in PG management.

## Methods

### Study design: Patient screening, inclusion criteria, endpoints

In this multicenter, retrospective case series, the characteristics and therapeutic strategies of PG as an extra-intestinal manifestation in IBD patients were analyzed. The study included data from patients across eight IBD tertiary care centers in Germany and Austria.

Patients with a confirmed diagnosis of IBD were identified according to standard criteria.

In all centers, the diagnosis of PG was established in accordance with the current ECCO guidelines and/or following dermatological consultation. In cases of diagnostic uncertainty, the diagnosis was further substantiated by histopathological evaluation of a biopsy specimen, which was performed in 8 of 50 patients (16%).

Data were collected using a standardized Case Report Form (CRF) across all participating centers. Inclusion criteria required a definitive IBD diagnosis and a documented occurrence of PG either in the past or during current treatment, with data collection concluding on March 31, 2025. Adequate documentation to complete the CRF and accurately depict disease progression was necessary for inclusion; otherwise, patients were excluded. Data were retrospectively gathered from patient charts.

IBD diagnoses had to be clinically, endoscopically, or histologically confirmed; patients with uncertain diagnoses were excluded. IBD activity at the onset of PG was defined using the partial Mayo score or the Harvey–Bradshaw Index. IBD activity during PG treatment was according to the physician’s global assessment. PG diagnoses were clinically determined, with optional histological confirmation not mandatory for study inclusion. PG had to be identified by the treating physician as an extra-intestinal manifestation of IBD.

Demographic and disease-specific data were collected, including prior surgeries and medications for IBD. PG characteristics were detailed, encompassing initial diagnosis, recurrences, complications, and IBD-specific medication at PG occurrence. Therapies initiated for PG treatment were characterized by duration, dosage, and therapeutic response, assessed by the treating physician as either resolution, defined as complete healing or resolution of PG, or lack of resolution, defined as incomplete healing or nonresponse. Each therapeutic attempt was recorded according to its timing as therapeutic stages. IBD activity was assessed as either in remission or not in remission according to the physician’s global assessment.

The reporting of this study conforms to the CARE guidelines (Consensus-based Clinical Case Reporting Guideline Development) for clinical case reporting.^15^ All patient details were de-identified for publication. The CARE checklist can be found in the Supplemental Material.

### Statistical analysis

Categorical variables are presented as absolute numbers and relative frequencies (*n*, %), and continuous variables as median with first and third quartiles (*Q*1, *Q*3). Groups were compared using the Mann–Whitney *U* test for continuous variables and Fisher’s exact test for categorical variables. All reported *p*-values are two-sided, and a significance level of 0.05 was considered significant. Univariable binary logistic regression analyses were performed to evaluate the association between PG resolution and individual demographic, clinical, and disease-related variables, including age, sex, IBD type, and clinical IBD remission. All statistical calculations were performed using SPSS Statistics for Windows, Version 29.0.0 (IBM Corporation, Armonk, NY, USA).

## Results

### Study population

A total of 50 patients from eight tertiary IBD centers were included in this cohort of patients with IBD and PG as an EIM. The data were collected between September 2024 and April 2025. The observation period extended from September 2007 to March 2025.

The cohort comprised a higher proportion of female patients (68%) with a median age of 43 (32, 56) years. CD was present in 58% of cases (29/50) and 42% suffered from UC (21/50). Regarding prior treatments, 50% of patients (25/50) had undergone surgical intervention, 68% of those received a stoma (17/25). 96% of patients (48/50) had already received IBD-specific therapy in their medical history. In the remaining two patients, PG occurred at the time of the initial diagnosis of IBD. Previous biologic therapy had been administered in 48% of patients (24/50), the majority of whom had received anti-TNF therapy (83%, 20/24); 50% (12/24) had been treated with more than one biologic agent. PG was most frequently localized to the lower extremities (52%, 26/50). Approximately 24% of patients (12/50) presented with peristomal PG, representing 71% of patients with an intestinal stoma (12/17). The remaining patients exhibited PG at other sites or had multifocal manifestations. Only four patients (8%) had a prior history of PG. In approximately half of the cohort (48%, 24/50 patients), additional EIMs were present alongside PG.

In the majority of patients (96%, 48/50), PG occurred at a median (*Q*1, *Q*3) of 9 (4, 13) years after the initial diagnosis of IBD. In one patient, PG developed 6 months prior to the diagnosis of IBD, while in another patient, the diagnosis of PG preceded the initial IBD diagnosis by 4 years.

In most cases, PG developed during ongoing IBD-specific therapy (78%, 39/50). Eleven out of 50 patients (22%) were on mesalazine, four patients (8%) received conventional immunosuppressive therapy with either prednisolone or azathioprine/methotrexate. Fifteen of 50 patients (30%) were undergoing active biologic therapy (anti-TNF, vedolizumab (VDZ), anti-IL-23, ustekinumab). In 4 out of 50 patients (8%), PG occurred under treatment with calcineurin inhibitors. Table 1 summarizes the demographic and clinical characteristics of the study population.

**Table 1.** Demographic and clinical characteristics of the study population: IBD patients with PG as an EIM.

Characteristics	IBD patients with PG (*n* = 50)
Age, yrs (median (*Q*1, *Q*3))	43 (32, 56)
Gender	
- Female (*n* (%))	34 (68)
- Male (*n* (%))	16 (32)
Disease type	
- CD (*n* (%))	29 (58)
- UC (*n* (%))	21 (42)
CD location (among respective patients)	
- Ileum isolated (*n* (%))	2 (7)
- Colon isolated (*n* (%))	10 (35)
- Ileocolic (*n* (%))	14 (48)
- Ileocolic + jejunum (*n* (%))	0 (0)
- Ileocolic + upper GIT (*n* (%))	3 (10)
Extent of UC (among respective patients)	
- Proctitis (*n* (%))	0 (0)
- Left-sided colitis (*n* (%))	6 (29)
- Pancolitis (*n* (%))	15 (71)
Previous surgery (*n* (%))	25 (50)
- Stoma (*n* (%))	17 (68)
- IPAA/pouch (*n* (%))	3 (12)
- Others (*n* (%))	5 (20)
Prior therapies (*n* (%))	48 (96)
- Biological experienced	24 (48)
- Anti-TNF (*n* (%))	20 (83)
- 1 Biological (*n* (%))	12 (50)
- 2–3 Biological (*n* (%))	9 (38)
- >3 Biological (*n* (%))	3 (13)
Localization of PG	
- Peristomal (*n* (%))	12 (24)
- Lower extremities (*n* (%))	26 (52)
- Other/multifocal/unknown (*n* (%))	12 (24)
Prior PG (*n* (%))	4 (8)
Concomitant EIM (*n* (%))	24 (48)
IBD therapy at initial diagnosis of PG	
- None (*n* (%))	11 (22)
- Prednisolone (*n* (%))	4 (8)
- Azathioprin/MTX (*n* (%))	4 (8)
- Mesalazine (*n* (%))	11 (22)
- Anti-TNF (*n* (%))	5 (10)
- Ustekinumab (*n* (%))	7 (14)
- Anti-IL-23 (*n* (%))	1 (2)
- JAK inhibitors (*n* (%))	1 (2)
- Vedolizumab (*n* (%))	2 (4)
- Calcineurin inhibitors (*n* (%))	4 (8)

CD, Crohn’s disease; EIM, extraintestinal manifestation; IBD, inflammatory bowel disease; IL, interleukin; IPAA, ileal pouch-anal anastomosis; JAK, Janus kinase; MTX, methotrexate; *n*, total number of patients; PG, pyoderma gangrenosum; *Q*1, lower (first) quartile; *Q*3, upper (third) quartile; TNF, tumor necrosis factor; UC, ulcerative colitis; yrs, years.

### Course of PG and IBD during therapy

In the above-described cohort of 50 patients with IBD and PG, complete healing of PG was achieved in 40 patients (80%) following initiation of a specific therapy with a median time to healing of 4 months (2.8 months). Among these 40 patients, the majority (78%, 31/40) were also in clinical remission of their underlying IBD. Five patients showed signs of active IBD, while in four cases the remission status of IBD was unclear.

Of the remaining 10 patients, 7 (14% of the cohort) demonstrated only partial improvement. Most of these patients (57%, 4/7) showed no signs of active IBD. The final three patients were refractory to all administered therapies and showed no clinical improvement of PG. None of these patients were in IBD remission at the time of PG onset or during treatment.

One female patient exhibited pronounced therapeutic refractoriness to multiple lines of treatments targeting both PG and CD. She underwent two courses of anti-TNF therapy (adalimumab and infliximab), anti-IL-23 therapy (risankizumab), JAK inhibition (upadacitinib), and calcineurin inhibition (ciclosporin A). A final attempt with cold plasma therapy also failed to induce a significant improvement in either PG or CD activity.

### Prednisolone showed limited effectiveness in the treatment of PG associated with IBD

**Figure 1.**
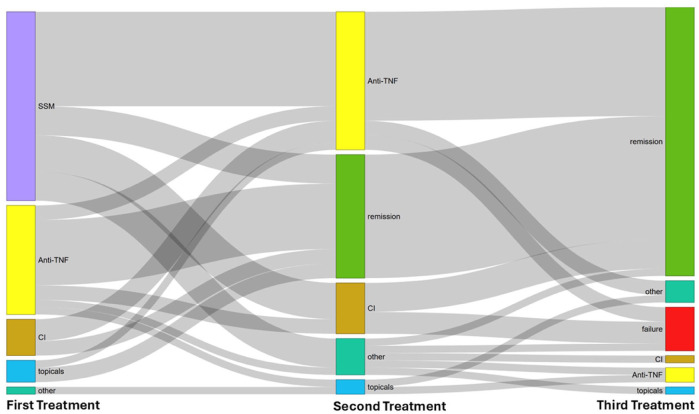
Frequency of therapeutics used during different attempts of PG therapy and their remission rates. CI, calcineurin inhibitor; PG, pyoderma gangrenosum; SSM, systemic steroid monotherapy.

More than half of the patients (52%, 26/50) received an initial therapeutic trial with systemic prednisolone as monotherapy, with a median initial dosage of 60 mg (range: 50–100 mg) for a median duration of 2 (1, 2) months (in three patients, the duration was unavailable; Figure 1). Complete healing of PG was achieved in only 3 out of 26 patients (11.5%) in 1, 1.5, and 2 months, only one of these patients was in clinical remission of the IBD. Due to the lack of clinical improvement, therapy escalation was required in the remaining patients. In many cases, prednisolone was later continued as part of a combination therapy or tapered over time, with different doses and reduction regimes.

### Anti-TNF therapy showed high efficacy in achieving clinical response of PG in IBD

**Figure 2.**
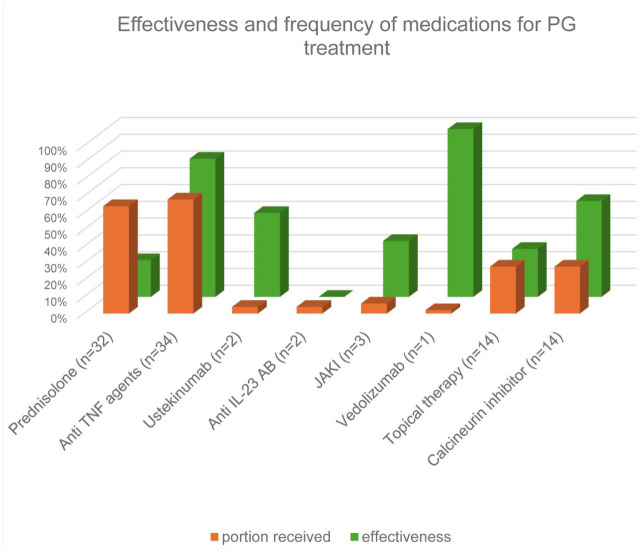
Summary of the treatments given for PG and the effectiveness of those medications. AB, antibody; JAKi, Janus kinase inhibitor; *n*, number of patients; PG, pyoderma gangrenosum; TNF, tumor necrosis factor.

The main therapeutic approach was anti-TNF agents. A total of 34 out of 50 patients (68%) received at least one anti-TNF agent. In 97% (33 of these 34) of patients, anti-TNF therapy was initiated as first- or second-line treatment (Figure 2). The most frequently used agent was infliximab, administered in 82% of cases (28/34 patients). 26% of patients (9/34) were treated with adalimumab. One female patient received golimumab as a fifth-line therapy.

The majority of these patients were female (68%, 23/34) with a median age of 42 (32, 54) years. Twenty patients (59%) had CD, while 14 (41%) were diagnosed with UC. Half of the patients (17/34) had undergone prior surgery, and among these, 12 (71%) had a stoma. Nearly half of patients (44%, 15/34) had previously been treated with at least one biologic agent, with 12 patients (35%) having received anti-TNF therapy. As observed in the overall cohort, PG was most commonly located on the lower extremities (56%, 19/34). Nine patients (26%) presented with peristomal PG.

Four patients (11%) were treated with more than one anti-TNF therapy for PG treatment due to an in-class switch. In two patients, initial therapy with infliximab was switched to adalimumab due to incomplete healing of PG. One patient received infliximab following an unsuccessful course of adalimumab. The remaining patient was treated with golimumab after failure of infliximab therapy.

Overall, a substantial proportion of patients—80% (27/34)—achieved complete healing of PG. Among those who did not respond to the initial anti-TNF agent, 3/4 patients (75%) achieved clinical improvement after switching to a second anti-TNF agent. The median time to complete healing under anti-TNF therapy was 5.5 (3, 8) months. At the time of complete PG healing, the majority of patients—79% (22/28)—were also in clinical remission of their underlying IBD. One female patient with a PG on the lower extremity, who had previously achieved complete remission under infliximab therapy, was successfully managed upon recurrence with topical infliximab administered as a gel after failure of adalimumab. Another female patient of this subcohort with a peristomal PG underwent a therapeutic attempt with sublesional injection of infliximab into the PG, which proved ineffective. Under systemic infliximab therapy, complete healing of the PG was subsequently achieved. Both therapeutic attempts have already been published as case reports.^16,17^

### Treatment with calcineurin inhibitors showed low efficacy in achieving clinical improvement of PG in the context of IBD

Overall, 13 out of 50 patients (26%) received systemic therapy with a calcineurin inhibitor (Figure 1). Tacrolimus was the most frequently used agent, administered in 11 out of 13 patients (85%), while ciclosporin A was given to 2 patients (15%). Resolution of PG was achieved in 5 of 13 patients (38%) treated with a calcineurin inhibitor after a median of 4 (4–5) months; 4 patients received tacrolimus and 1 ciclosporin A. In three patients who initially received a calcineurin inhibitor as first-line therapy, switching to anti-TNF therapy subsequently led to complete healing of PG.

In addition, five patients underwent a trial of topical calcineurin inhibitors for the treatment of PG: ciclosporin A was used in three cases and tacrolimus in two. In both tacrolimus-treated cases, the agent was applied as a first-line therapy and resulted in the resolution of PG. One of these patients also received concomitant systemic prednisolone therapy, initiated at 60 mg daily.

Topical application of ciclosporin A led to resolution in one of the three cases. In the remaining two patients, ciclosporin A was used as a first- or second-line treatment but failed to induce resolution. In both cases, systemic biologic therapy with adalimumab or risankizumab was subsequently initiated, each resulting in the resolution of PG.

### Outcomes of JAK inhibition and other biologic agents beyond anti-TNF therapy in PG

JAK inhibitors were used in a total of four cases (8%), with upadacitinib being the agent administered in all instances. Complete healing of PG was achieved in one patient (25%) as a second-line treatment. In this case, upadacitinib was initiated after first-line treatment as prednisolone and infliximab had failed. Complete healing of PG was observed after 4 months, accompanied by clinical remission of UC. One patient received upadacitinib as a second-line therapy following unsuccessful treatment with prednisolone. Subsequent escalation to a calcineurin inhibitor also failed to induce complete healing of PG. Another patient was treated with upadacitinib as a third-line option. Prior therapies included intravenous immunoglobulins (first-line) and topical infliximab gel, both of which failed to achieve complete healing of PG. This was ultimately achieved under treatment with golimumab, after failure of infliximab and prior failure of adalimumab. Upadacitinib was continued for the management of CD. The remaining patient received upadacitinib as a fourth-line therapy but showed no clinical response with regard to PG and exhibited marked CD activity. Despite multiple therapeutic attempts, neither resolution of PG nor induction of remission of CD has been achieved to date.

Ustekinumab, an anti-IL-12/23 inhibitor, was used in two cases (4%). In one patient, complete healing of PG affecting the lower extremity was achieved after 1 month of treatment. Prior therapies with prednisolone (first-line) and infliximab (second-line) had failed. At the time of PG resolution, the patient’s CD was also in clinical remission. The second patient also had CD and presented with multifocal PG. Ustekinumab was initiated as second-line therapy following unsuccessful treatment with prednisolone, but it did not result in complete healing of PG. As the patient remained in remission with respect to CD, no further systemic therapy was initiated at that time. A subsequent attempt with topical dapsone led to partial healing of PG.

Risankizumab, a selective IL-23 inhibitor, was used in two cases (4%), but failed to induce resolution of PG in either case. In one patient, risankizumab was administered as second-line therapy following unsuccessful treatment with prednisolone. In the second case, risankizumab was used as a fourth-line option in the previously described therapy-refractory patient who had undergone multiple prior treatment attempts. Both patients exhibited active underlying disease at the time of PG manifestation and during risankizumab therapy.

One female patient with UC received a trial of VDZ for PG located on the lower extremity. VDZ was initiated following failure of prior therapy with adalimumab. Complete healing of PG was achieved after 3 months of treatment, and the patient’s UC was also in clinical remission.

Figure 2 summarizes the treatments given for PG and the number of patients improving on those medications.

### Outcomes by PG localization

The majority of patients (26/50, 52%) presented with PG localized on the lower extremities (Table 2). In 80% of these patients (23/26), complete healing of PG was achieved, with a median time to resolution of 4 (2, 8) months.

**Table 2.** Demographic and clinical characteristics and therapeutic response of PG in patients with IBD: comparison of PG localization (peristomal, lower extremities, multifocal/other/unknown).

Demographic and clinical characteristics	Peristomal (*n* = 12)	Lower extremities (*n* = 26)	Multifocal/other/unknown (*n* = 12)
Age, yrs (median (*Q*1, *Q*3))	45 (29, 67)	40 (32, 53)	46 (30, 57)
Gender			
- Female (*n* (%))	9 (75)	15 (58)	10 (83)
- Male (*n* (%))	3 (25)	11 (42)	2 (17)
Disease type			
- CD (*n* (%))	11 (92)	13 (50)	7 (58)
- UC (*n* (%))	1 (8)	13 (50)	5 (42)
Prior therapies			
- Biological experienced (*n* (%))	11 (92)	8 (31)	7 (58)
- Anti-TNF (*n* (%))	10 (83)	6 (26)	4 (33)
Treatment outcome			
- PG resolution (*n* (%))	10 (83)	23 (88)	7 (58)
- Time to resolution, months (median (*Q*1, *Q*3))	3 (3, 9)	4 (2, 8)	4 (3, 8)
- Clinical remission of IBD (*n* (%))	8 (80)	20 (87)	3/7 (43)

CD, Crohn’s disease; IBD, inflammatory bowel disease; *n*, total number of patients; PG, pyoderma gangrenosum; *Q*1, lower (first) quartile; *Q*3, upper (third) quartile; TNF, tumor necrosis factor; UC, ulcerative colitis; yrs, years.

Twelve patients (24%) presented with peristomal PG. Most of these patients (92%, 11/12) had been previously treated with at least one biologic, with the majority also having received anti-TNF therapy (83%, 10/12). In most of these patients, complete healing of PG was achieved (83%, 10/12) after a median of 3 (3, 9) months. At the time of PG healing, 8 out of 12 patients (67%) were in clinical remission of their IBD, while 2 patients had active disease, and in the remaining 2 cases, the remission status was unclear.

There were no statistically significant differences between the two subgroups regarding gender distribution (*p* = 0.472) or median age (*p* = 0.631). With respect to disease type, patients with peristomal PG showed a significantly higher proportion of CD compared to UC (*p* = 0.027). Similarly, patients with peristomal PG had a significantly higher rate of previous biologic exposure (*p* = 0.001), particularly anti-TNF therapy (*p* = 0.001). The rate of resolution was comparable in both subgroups, with no significant difference related to PG localization (*p* = 0.643). Likewise, no statistically significant difference was found between the groups in terms of time to resolution (*p* = 0.773).

The remaining 12 out of 50 patients (24%) presented with either multifocal PG or PG at other anatomical sites and were grouped together for analysis. This heterogeneous subgroup also consisted predominantly of female patients (83%, 10/12) with a median age of 46 (30, 57) years. Seven patients (58%) had CD, while five patients (42%) had UC. Prior biologic therapy was documented in 7 out of 12 patients (58%), with 4 patients (33%) having received anti-TNF therapy in the past. Complete healing of PG was achieved in 7 out of 12 patients (58%) after a median of 4 (3, 8) months. At the time of PG resolution, three out of the seven patients (43%) were also in confirmed clinical remission of their underlying IBD.

### Sex-specific clinical characteristics and treatment outcomes

Most of the cohort consisted of female patients, accounting for 68% (34/50), with a median age of 42 (32, 56) years. Among them, 65% (22/34) had CD, and 35% (12/34) had UC. A prior biologic therapy had been administered to 18/34 patients (53%), including anti-TNF therapy in 14/34 cases (41%). Complete healing of PG was achieved in 25/34 female patients (74%) after a median of 5 (3, 8) months, with 18 of these 25 patients (72%) also in clinical remission of their underlying IBD at the time of PG resolution. The remaining 16 patients (32%) were male, with a median age of 46 (38, 63) years. This subgroup exhibited a higher proportion of UC (56%, 9/16) compared to CD (44%, 7/16). Six of these patients (38%) had previously received biologic therapy, all of whom had been treated with anti-TNF therapy. A total of 15/16 male patients (94%) experienced complete healing of PG after a median of 3 (2, 6) months (data available for 14/15), with 13 of these 15 patients (87%) also in clinical remission of their IBD at the time of PG resolution.

Comparison between male and female subgroups revealed no statistically significant differences in median age (*p* = 0.417), distribution of CD or UC (*p* = 0.222), prior biologic therapy (*p* = 0.372), or anti-TNF therapy (*p* = 1). While the overall PG resolution rate did not differ significantly between sexes (*p* = 0.138), the median time to complete healing was approximately 2 months shorter in male patients (*p* = 0.049).

### Clinical characteristics and treatment outcomes in CD and UC patients with PG

CD was the leading underlying IBD in our cohort (58%; 29/50, Table 3). Most patients exhibited either isolated colonic involvement or an ileocolonic disease pattern (83%). The CD subgroup was predominantly female (76%, 22/29) with a median age of 42 (33, 57) years. At the time of PG onset, 59% of patients (17/29) had prior exposure to biologic therapy, 48% (14) had exposure to anti-TNF therapy. Resolution of PG was achieved in 83% of cases (23/29) after a median of 4 (2, 8) months. Among those with available remission data, 86% (18/21) were also in clinical remission of their CD at the time of PG resolution.

**Table 3.** Demographic and clinical characteristics and therapeutic resolution of PG in IBD: comparison of CD and UC.

Demographic and clinical characteristics and treatment outcome	CD (*n* = 29)	UC (*n* = 21)
Age, yrs (median (*Q*1, *Q*3))	42 (33, 57)	44 (32, 56)
Gender		
- Female (*n* (%))	22 (76)	12 (57)
- Male (*n* (%))	4 (24)	9 (43)
CD location (among respective patients)		
- Ileum isolated (*n* (%))	2 (7)	n/a
- Colon isolated (*n* (%))	10 (35)	n/a
- Ileocolic (*n* (%))	14 (48)	n/a
- Ileocolic + jejunum (*n* (%))	0 (0)	n/a
- Ileocolic + upper GIT (*n* (%))	3 (10)	n/a
Extent of UC (among respective patients)		
- Proctitis (*n* (%))	n/a	0 (0)
- Left-sided colitis (*n* (%))	n/a	6 (29)
- Pancolitis (*n* (%))	n/a	15 (71)
Prior therapies		
- Biological experienced (*n* (%))	17 (59)	7 (33)
- Anti-TNF (*n* (%))	14 (48)	5 (24)
Localization of PG		
- Peristomal (*n* (%))	11 (38)	1 (5)
- Lower extremities (*n* (%)	13 (45)	13 (62)
- Other/multifocal/unknown (*n* (%))	5 (17)	7 (33)
Treatment outcome		
- PG resolution (*n* (%))	23 (83)	17 (83)
- Time to resolution, months (median (*Q*1, *Q*3))	4 (2, 8)	4 (3, 7)
- Clinical remission of IBD (*n* (%))^a^	12 (57)	12/17 (71)

a Available data from 21/23 patients with CD and PG resolution.

CD, Crohn’s disease; IBD, inflammatory bowel disease; *n*, total number of patients; n/a, not applicable; PG, pyoderma gangrenosum; *Q*1, lower (first) quartile; *Q*3, upper (third) quartile; TNF, tumor necrosis factor; UC, ulcerative colitis; yrs, years.

UC was present in 21 patients (42%), with pancolitis being the predominant disease pattern (71%). This subgroup was predominantly female (57%, 12/21) and had a median age of 44 (32, 56) years. Prior biologic therapy before the onset of PG was documented in 33% of patients (7/21), with 24% (5) having received anti-TNF therapy. Resolution of PG occurred in 81% of patients (17/21) after a median of 4 (3, 7) months, based on available data for 14 out of 17 patients. Among these, 71% (12/17) were in clinical remission of their UC at the time of PG resolution.

Both subgroups demonstrated a comparable female predominance (*p* = 0.222), with no statistically significant differences observed in median age (*p* = 0.969), prior biologic therapy (*p* = 0.093), or prior anti-TNF therapy (*p* = 0.139). Similarly, there was no statistically significant difference in PG resolution rates between patients with CD and those with UC (*p* = 1.0) and in the median time to PG resolution (*p* = 1).

Univariable logistic regression analyses were performed to identify prognostically relevant factors associated with PG resolution.

In univariable logistic regression analysis, clinical IBD remission was significantly associated with PG resolution (odds ratio 5.17, 95% confidence interval 1.19–22.40; *p* = 0.03), whereas age, sex, and disease type were not (Table 4). The association between PG healing and IBD remission was additionally assessed using Fisher’s exact test, confirming a statistically significant association (*p* = 0.007).

**Table 4.** Univariable logistic regression analysis of factors associated with PG resolution.

Variable	OR	95% Confidence interval	*p*-Value
Age (per year)	0.99	0.93–1.02	0.19
Female sex	0.19	0.02–1.61	0.13
Crohn’s disease	0.90	0.22–3.70	0.89
Clinical IBD remission at the time of PG resolution	5.17	1.19–22.40	0.03

IBD, inflammatory bowel disease; OR, odds ratio; PG, pyoderma gangrenosum.

## Discussion

The treatment of PG in patients with IBD remains challenging. Evidence on therapeutic efficacy is limited and largely based on older cohort studies, small case series, and a single randomized placebo-controlled trial evaluating infliximab, which included only a small number of IBD patients. Comparative data on treatment strategies, particularly in patients with multiple prior IBD therapies, are lacking. Moreover, PG is rare even in specialized centers, limiting the feasibility of controlled trials. Against this background, our case series provides relevant real-world data for clinical practice.

A key finding of our study is the low response rate to systemic corticosteroids, despite their recommendation as first-line therapy in several guidelines, including the German S3 and European ECCO guidelines.^2,4^ By contrast, the high response rates observed with anti-TNF therapy are consistent with the limited available literature, which mainly consists of retrospective studies and case reports.

To date, only one double-blind, placebo-controlled randomized trial has assessed infliximab in PG, including 30 patients, 19 of whom had IBD. At week 2, significant clinical improvement was observed in 6/13 patients treated with infliximab compared with 1/17 receiving placebo (46% vs 6%, *p* = 0.025). During the subsequent open-label phase, 69% of patients showed clinical improvement, while complete resolution at week 6 was achieved in 21% (6/29); 31% showed no response.^12^ A systematic review from 2017, including 9 interventional and 13 non-interventional studies (2854 patients), evaluated anti-TNF therapy for EIMs of IBD. In the PG subgroup, resolution rates ranged from 21% to 25% in interventional studies and 92%–100% in non-interventional studies,^3^ consistent with our findings. However, given the observational design, high response rates may partly reflect selection bias and the high proportion of anti-TNF-treated patients.

Overall prognosis of PG in IBD appears favorable. In our cohort, 80% of patients achieved remission, in line with a single-center retrospective study reporting remission in 75% of IBD-associated PG cases at 12 months.^13^ Most patients required more than one treatment line to achieve resolution, consistent with previous reports showing that over 70% of patients need multiple therapies.^14^

PG healing usually requires several months. In our cohort, the median healing time was 4 months, shorter than the up to 12 months reported in the literature.^18^ This may be explained by non-standardized outcome definitions, heterogeneous follow-up intervals, and advances in IBD treatment over the long study period (2007–2025), including earlier and more intensive use of biologic agents. Shorter healing times have likewise been reported in a recent meta-analysis of IBD-associated PG.^19^

Peristomal PG accounted for 24% of cases in our cohort. Peristomal PG has been reported in approximately 0.6% of all patients with intestinal stomas,^20^ although higher proportions among PG cases have been described in prospective studies.^21^ Differences likely reflect variation in study populations and referral patterns. A recent single-center cohort study reported frequent use of intralesional and oral corticosteroids in both peristomal and non-peristomal PG.^22^ By contrast, intralesional therapy was rarely used in our cohort and was applied in only one patient without success,^17^ reflecting differences in national treatment practices.

PG healing was significantly associated with remission of underlying IBD (*p* = 0.007). This is consistent with findings reported in the literature, in which PG healing and IBD remission have been shown to be associated.^13,23^ However, due to the small sample size and non-stringent definition of IBD remission, this finding should be interpreted cautiously.

The main limitations of this study include its retrospective design, limited sample size, lack of standardized PG diagnostic criteria, absence of long-term follow-up and recurrence data, and inability to adjust for confounding factors. Nevertheless, given the scarcity of data on PG in IBD, this study provides meaningful insights into treatment strategies and disease course in routine clinical practice.

## Conclusion

In conclusion, PG in IBD patients most commonly affects the lower extremities, although peristomal involvement is frequent and represents a particular therapeutic challenge. Despite limited evidence, current recommendations and the results of this study suggest that anti-TNF therapy remains an effective treatment option for IBD-associated PG, whereas response to systemic corticosteroids appears limited. Future studies should focus on prospective trials or large, well-controlled retrospective cohorts to better inform evidence-based clinical decision-making.

## Supplemental Material

sj-pdf-1-tag-10.1177_17562848261461012 – Supplemental material for International collaborative experiences in managing pyoderma gangrenosum in patients with inflammatory bowel disease Supplemental material, sj-pdf-1-tag-10.1177_17562848261461012 for International collaborative experiences in managing pyoderma gangrenosum in patients with inflammatory bowel disease by Jan Guse, Andreas Blesl, Philip Esters, Katja Matthes, Renate Schmelz, Carsten Schmidt, Julia Wanzl, Elisabeth Schnoy, Niels Teich, Viktoria Hentschel, Andreas Stallmach, Jessica Rueddel and Kathleen Lange in Therapeutic Advances in Gastroenterology

## Appendix

### Abbreviations

AB antibody

CD Crohn’s disease

CRF case report form

EIM extraintestinal manifestation

IBD inflammatory bowel disease

IL-23 interleukin-23

IPAA ileal pouch anal anastomosis

JAKi Janus kinase inhibitor

MTX methotrexate

PG pyoderma gangrenosum

TNF tumor necrosis factor

UC ulcerative colitis

VDZ vedolizumab

## Declarations

**Ethics approval and consent to participate:** The study was approved by the local ethics committee under approval number/ID 2024-3514. The requirement for informed consent was waived because of the retrospective nature of the study and the use of pseudonymized data.

**Consent for publication:** The study was approved by the local ethics committee, which waived the requirement for informed consent due to the retrospective design and the use of pseudonymized data.

**Availability of data and materials:** The data supporting the findings of this study are not publicly available due to data protection regulations and patient confidentiality but are available from the corresponding author upon reasonable request.*
