# Supplementary material for: International collaborative experiences in managing pyoderma gangrenosum in patients with inflammatory bowel disease
Source: Ther Adv Gastroenterol. 2026 Jun 29;19:17562848261461012. doi: 10.1177/17562848261461012 (PMC13319564; doi:10.1177/17562848261461012)
Supplement: sj-pdf-1-tag-10.1177_17562848261461012 – Supplemental material for International collaborative experiences in managing pyoderma gangrenosum in patients with inflammatory bowel disease [file sj-pdf-1-tag-10.1177_17562848261461012.pdf]

CARE case report guidelines

CARE Checklist of information to include when writing a case report

(cc) BY-NC-ND

Topic	Item	Checklist item description	Reported on Line
Title	1	The diagnosis or intervention of primary focus followed by the words "case report"	NA
Key Words	2	2 to 5 key words that identify diagnoses or interventions in this case report, including "case report"	NA
Abstract (no references)	3a	Introduction: What is unique about this case and what does it add to the scientific literature?	page 3
	3b	Main symptoms and/or important clinical findings	page 3
	3c	The main diagnoses, therapeutic interventions, and outcomes	page 3
	3d	Conclusion—What is the main "take-away" lesson(s) from this case?	page 3
Introduction	4	One or two paragraphs summarizing why this case is unique (**may include references**)	page 5, line 92-104
Patient Information	5a	De-identified patient specific information.	page 7, line 135-136
	5b	Primary concerns and symptoms of the patient.	page 8-9
	5c	Medical, family, and psycho-social history including relevant genetic information	NA
	5d	Relevant past interventions with outcomes	page 9-11, table 1
Clinical Findings	6	Describe significant physical examination (PE) and important clinical findings.	NA
Timeline	7	Historical and current information from this episode of care organized as a timeline	NA
Diagnostic Assessment	8a	Diagnostic testing (such as PE, laboratory testing, imaging, surveys).	NA
	8b	Diagnostic challenges (such as access to testing, financial, or cultural)	NA
	8c	Diagnosis (including other diagnoses considered)	page 8-11, table 1
	8d	Prognosis (such as staging in oncology) where applicable	NA
Therapeutic Intervention	9a	Types of therapeutic intervention (such as pharmacologic, surgical, preventive, self-care)	page 11-23, figure 1 and 2
	9b	Administration of therapeutic intervention (such as dosage, strength, duration)	page 11-23
	9c	Changes in therapeutic intervention (with rationale)	page 11-23, figure 1 and 2
Follow-up and Outcomes	10a	Clinician and patient-assessed outcomes (if available)	page 11-23, figure 1 and 2
	10b	Important follow-up diagnostic and other test results	NA
	10c	Intervention adherence and tolerability (How was this assessed?)	page 11-23, clinical
	10d	Adverse and unanticipated events	page 11-23
Discussion	11a	A scientific discussion of the strengths AND limitations associated with this case report	page 24-25
	11b	Discussion of the relevant medical literature **with references**.	page 24-25
	11c	The scientific rationale for any conclusions (including assessment of possible causes)	page 24-25
	11d	The primary "take-away" lessons of this case report (without references) in a one paragraph conclusion	page 25-26
Patient Perspective	12	The patient should share their perspective in one to two paragraphs on the treatment(s) they received	NA
Informed Consent	13	Did the patient give informed consent? Please provide if requested	Yes ☐ No ☒
